# Association of Time–Varying Intensity of Ventilation With Mortality in Patients With COVID−19 ARDS: Secondary Analysis of the PRoVENT–COVID Study

**DOI:** 10.3389/fmed.2021.725265

**Published:** 2021-11-18

**Authors:** Michiel T. U. Schuijt, David M. P. van Meenen, Ignacio Martin–Loeches, Guido Mazzinari, Marcus J. Schultz, Frederique Paulus, Ary Serpa Neto

**Affiliations:** ^1^Department of Intensive Care, Amsterdam UMC, Location AMC, Amsterdam, Netherlands; ^2^Department of Clinical Medicine, Trinity Centre for Health Sciences, Multidisciplinary Intensive Care Research Organization (MICRO), St James's Hospital, Dublin, Ireland; ^3^Department of Anaesthesiology, Hospital Universitario y Politécnico la Fe, Valencia, Spain; ^4^Mahidol Oxford Tropical Medicine Research Unit (MORU), Mahidol University, Bangkok, Thailand; ^5^Nuffield Department of Medicine, University of Oxford, Oxford, United Kingdom; ^6^ACHIEVE, Centre of Applied Research, Amsterdam University of Applied Sciences, Faculty of Health, Amsterdam, Netherlands; ^7^Australian and New Zealand Intensive Care Research Centre (ANZIC–RC), Monash University, Melbourne, VIC, Australia; ^8^Department of Intensive Care, Austin Hospital, Melbourne, VIC, Australia; ^9^Data Analytics Research and Evaluation (DARE) Centre, Austin Hospital, Melbourne, VIC, Australia; ^10^Department of Critical Care, Melbourne Medical School, University of Melbourne, Melbourne, VIC, Australia; ^11^Department of Critical Care Medicine, Hospital Israelita Albert Einstein, São Pãulo, Brazil

**Keywords:** coronavirus disease 2019, acute respiratory distress syndrome, driving pressure, mechanical power, mortality

## Abstract

**Background:** High intensity of ventilation has an association with mortality in patients with acute respiratory failure. It is uncertain whether similar associations exist in patients with acute respiratory distress syndrome (ARDS) patients due to coronavirus disease 2019 (COVID−19). We investigated the association of exposure to different levels of driving pressure (ΔP) and mechanical power (MP) with mortality in these patients.

**Methods:** PRoVENT–COVID is a national, retrospective observational study, performed at 22 ICUs in the Netherlands, including COVID−19 patients under invasive ventilation for ARDS. Dynamic ΔP and MP were calculated at fixed time points during the first 4 calendar days of ventilation. The primary endpoint was 28–day mortality. To assess the effects of time–varying exposure, Bayesian joint models adjusted for confounders were used.

**Results:** Of 1,122 patients included in the PRoVENT–COVID study, 734 were eligible for this analysis. In the first 28 days, 29.2% of patients died. A significant increase in the hazard of death was found to be associated with each increment in ΔP (HR 1.04, 95% CrI 1.01–1.07) and in MP (HR 1.12, 95% CrI 1.01–1.36). In sensitivity analyses, cumulative exposure to higher levels of ΔP or MP resulted in increased risks for 28–day mortality.

**Conclusion:** Cumulative exposure to higher intensities of ventilation in COVID−19 patients with ARDS have an association with increased risk of 28–day mortality. Limiting exposure to high ΔP or MP has the potential to improve survival in these patients.

**Clinical Trial Registration:** www.ClinicalTrials.gov, identifier: NCT04346342.

## Introduction

The coronavirus disease 2019 (COVID−19) is an infection caused by the highly contagious Severe Acute Respiratory Coronavirus−2 (SARS–CoV−2), of which the first outbreak was reported in Wuhan, China in the beginning of December, 2019 (1). Worldwide, healthcare workers are faced with surges of infected patients who need hospitalization and eventually admission to an intensive care unit (ICU) because of need for invasive ventilation. The care of coronavirus disease 2019 (COVID−19) patients receiving ventilation is challenging and the outcomes are poor (2). Mortality rates as high as 50% have been reported in invasively ventilated COVID−19 patients that develop acute respiratory distress syndrome (ARDS) (2).

Different ventilation strategies have been studied in the setting of acute respiratory failure, and evidence from randomized clinical trials supports the use of ventilation with a low tidal volume (V_T_) and a low plateau pressure to decrease the risk of ventilator–induced lung injury (VILI) in patients with acute respiratory distress syndrome (ARDS) (3–5). There is increasing interest in limiting exposure to driving pressure (ΔP) and mechanical power (MP) in invasively ventilated patients (6–8). ΔP, the pressure applied by the ventilator to support the delivery of tidal volume, (7) is a surrogate for cyclic lung strain (9). MP, a parameter that combines several ventilatory variables, including respiratory rate (RR), V_T_, flow and ΔP, represents the amount of energy transferred from the ventilator to the respiratory system (8, 10). Associations of ΔP and MP with outcomes in patients receiving mechanical ventilation is well described (7, 8, 11–19).

A potential limitation of earlier studies is the inclusion of patients with spontaneous efforts during ventilation (18, 20). Even if appropriately adjusted for resistance, flow, and chest wall elastance, any estimate of these variables during spontaneous efforts would reflect both the ventilator's contribution and respiratory muscle activity, and thus would not represent the total energy imparted during inflation (20). Also, mechanical ventilation is a dynamic process, and thus far only one investigation studied the cumulative effect of ΔP and MP beyond baseline (12). To date, there have been no studies that assessed the impact of intensity of ventilation on outcome of COVID−19 patients with ARDS. To ascertain the effect of time–varying exposure to different levels of ΔP and MP on 28–day mortality in COVID−19 patients with ARDS, we conducted a secondary analysis of a national multicenter investigation, named the “PRactive of VENTilation in COVID−19” (PRoVENT–COVID) study. Our objectives were to estimate the effects of different levels of ΔP and MP over the first 4 days of ventilation on 28–day mortality in COVID−19 patients with ARDS, and whether there was a cumulative effect of exposure over time. We hypothesized that intensity of ventilation has an association with mortality in patients with COVID−19 ARDS, in a similar way as has been described in patients with ARDS from another cause.

## Methods

### Study Design and Participants

This is a preplanned secondary analysis of the “Practice of VENTilation in COVID−19” (PRoVENT–COVID) study, an investigator–initiated, multicenter, observational cohort study in patients with COVID−19 ARDS undertaken at 22 ICUs during the first 3 months of the pandemic in the Netherlands (21–23). The protocol of the PRoVENT–COVID study was prepublished, (21) and the statistical analysis plan for the current analysis, finalized before assessing the database, is available online (22). The institutional review boards of each participating center approved the study protocol, and need for individual patient informed consent was waived based on the observational nature of the study. Study sites were recruited through direct contact by members of the steering committee of the PRoVENT–COVID study. Study coordinators contacted the local doctors, trained and helped the data collectors, and monitored the study according to the International Conference on Harmonization Good Clinical Practice–guidelines. Integrity and timely completion of data collection was ensured by the study coordinators.

Consecutive patients aged 18 yr or older were eligible for participation if they were admitted to one of the participating ICUs and had received invasive mechanical ventilation for COVID−19 ARDS. The PRoVENT–COVID study itself had no exclusion criteria—for the current analysis we excluded patients if they had spontaneous breathing activity in more than half the observations, or when life status was unknown at day 28.

### Procedures and Outcome

Demographics and data regarding premorbid diseases and home medication were collected at baseline. In the first hour of invasive ventilation, and every 8 h thereafter at fixed time points ventilator settings and parameters were collected up to day 4. Since plateau pressure was not recorded in the current study, all measurements of dynamic ΔP were calculated as peak inspiratory pressure (P_peak_) minus positive end–expiratory pressure. Dynamic MP was calculated as 0.098 ^*^ RR ^*^ VT ^*^ [P_peak_ – (0.5 × dynamic ΔP)]. Both variables were calculated only considering moments without evidence of spontaneous breathing (additional information in eMethods in Supplementary Information). The Berlin definition for ARDS was used for classification of severity as mild, moderate and severe (24).

The primary outcome was 28–day mortality. In secondary analyses, we investigated whether the strength of association between intensity of ventilation and 28–day mortality changed over time. In addition, we quantified the effect of cumulative response, and we examined whether ARDS severity class changed the effects of time–varying ΔP and MP on 28–day mortality.

### Analysis Plan

For the final assessment, patients receiving ventilation without evidence of spontaneous breathing at less than 50% of the available timepoints were identified and deselected (Supplementary Information **eMethods**). Continuous variables were reported as median (25th – 75th percentile) and compared with Wilcoxon rank–sum tests, and categorical variables as number and percentage, and compared with Fisher exact tests. The following variables were considered for adjustment in all models described below: age, gender, body mass index, PaO_2_ to FiO_2_ ratio, plasma creatinine, medical history of hypertension, heart failure, diabetes, chronic kidney disease, chronic obstructive pulmonary disease, active hematological neoplasia and/or active solid tumor, use of angiotensin converting enzyme inhibitors, use of angiotensin II receptor blockers, use of a vasopressor or inotropes, fluid balance, arterial pH, mean arterial pressure, and heart rate. These baseline covariates were selected according to clinical relevance and as used in previous study (23). Finally, all continuous variables were standardized to interpret their effect on outcome in standard deviation units.

To estimate the association of subject–specific longitudinal profiles of either ΔP and MP with 28–day mortality, we used Bayesian joint models with shared random effects and adjusted for the covariates described above (25, 26). The repeated values of ΔP and MP (a maximum of 13 measurements) were included as time–varying exposure variables. Natural cubic splines were used in both the fixed–effects and random–effects models to account for the non-linearity of the longitudinal exposure profiles. To investigate whether the association between ΔP and MP and 28–day mortality changed over time, p–splines were included in an interaction term and presented in time–varying plots. Estimation of Bayesian joint model was done considering 28,000 iterations, 3,000 adapt, 3,000 burn–in, and 50 of thinning. Model diagnostics were done by visual inspection of the diagnostic plots. These results were presented as hazard ratios with corresponding 95% credible intervals (CrI).

To further expand the findings of the original model, 3 additional analyses were performed. First, the model described above was expanded to a multivariate joint model. In addition to the baseline covariates, the following time–varying covariates were included: daily use of prone positioning and daily use of inotropes or vasopressors. Second, the calculation of dynamic ΔP and MP were further restricted to moments when a neuromuscular blocking agent was administered, thereby decreasing the chance of including moments at which a patient was having spontaneous breathing activity. Third, patients with missing data in their 28–day vital status were not excluded but assessed in 2 different scenarios: i. best–case scenario (these patients were all considered alive at day 28); and ii. worst–case scenario (these patients were all considered to have died before or at day 28).

Three sensitivity analyses were added. First, to quantify the effect of cumulative exposure, we estimate the association between the percentage of moments with high ΔP and MP and 28–day mortality. The cut–off used to determine high ΔP was 15 cm H_2_O; the cut–off used for high MP was 17 J/min (7, 8, 12, 27). Second, we investigated the relationship between cumulative dose and 28–day mortality using the area under the ΔP and MP time curve above the thresholds described above divided by the number of hours of exposure, as a measure of dose. Using this definition, 1 cm H_2_O or 1 J/min of dose describes that a patient's average ΔP and MP were 1 cmH_2_O or 1 J/min per mL/cm H_2_O above the thresholds described for the duration of the exposure window. Third, we investigated the impact of time–weighted average ΔP and MP calculated as the area under the ΔP and MP time curve divided by the number of hours of exposure. For these 3 exposures, the impact on outcome was assessed using (shared–frailty) Cox proportional hazard models adjusted by the covariates described above.

The models were reassessed in a subgroup analysis according to ARDS severity at baseline (24). The models were repeated, considering an interaction between ΔP and MP and ARDS severity at baseline. The amount of missing data is <3% for the variables of interest, as shown in the Appendix (Supplementary Information Table 1). For the final models, missing data in covariates were imputed by the median due to the low number of missing, and in the repeated measurements of ΔP and MP over the days a linear imputation was used (Supplementary Information Figure 1). All analyses were performed using R version 4.0.2 (R Foundation for Statistical Computing), and a *p* < 0.05 was considered significant. *P* values for the Bayesian models were calculated as the tail probabilities using the formula 2 x min{P(θ > 0), P(θ <0)}, with θ denoting the corresponding regression coefficient from the survival submodel.

## Results

From March 1, 2020, through June 1, 2020, 31 ICUs were invited for participation in the PRoVENT–COVID study, and 22 met inclusion criteria. A total of 1,340 individuals were screened. Of the 1,122 invasively ventilated COVID−19 patients, 734 (66.6%) were included in the current analysis (Supplementary Information Figure 2). Reasons for exclusion were evidence for presence of spontaneous breathing activity in more than 50% of available timepoints of data collection (*n* = 368), and unknown life status at day 28 (*n* = 20). Demographic characteristics and ventilation characteristics are presented in Table 1. The patients had a median age of 65 yr [IQR 57–72] and 194 (26%) of them were women. 8.9% of the patients had mild ARDS, and 57.8% and 33.4% moderate or severe ARDS, respectively. The most prevalent premorbid conditions were hypertension and diabetes. 29.2% of the patients died within the first 28 days of follow–up. Additional clinical outcomes and use of rescue therapies are presented in the Appendix (Supplementary Information Table 2).

**Table 1:** Baseline patient characteristics and outcomes.

	**Participants** **(*n* = 734)**
Age, years	65 (57–72)
Male gender–no (%)	540 (73.6)
Body mass index, kg/m^2^	27.8 (25.4–31.1)
Transferred under invasive ventilation	109 (14.9)
Days between intubation and admission	0.0 (0.0–0.0)
Use of non–invasive ventilation prior to intubation–no (%)	66/661 (10.0)
Duration of non–invasive ventilation, hours	6 (2–14)
Chest CT scan performed–no (%)	254/715 (35.5)
Lung parenchyma affected–no (%)	
0%	9/254 (3.5)
25%	77/254 (30.3)
50%	80/254 (31.5)
75%	71/254 (28.0)
100%	17/254 (6.7)
Chest X–ray performed–no (%)	397/455 (87.3)
Quadrants affected–no (%)	
1	28/395 (7.1)
2	97/395 (24.6)
3	117/395 (29.6)
4	153/395 (38.7)
Severity of ARDS–no (%)	
Mild	65 (8.9)
Moderate	424 (57.8)
Severe	245 (33.4)
Co–existing disorders–no (%)	
Hypertension	247 (33.7)
Heart failure	27 (3.7)
Diabetes	163 (22.2)
Chronic kidney disease	29 (4.0)
Baseline creatinine, μmol/L^*^	78 (62–100)
Liver cirrhosis	2 (0.3)
Chronic obstructive pulmonary disease	62 (8.4)
Active hematological neoplasia	9 (1.2)
Active solid neoplasia	21 (2.9)
Neuromuscular disease	3 (0.4)
Immunosuppression	15 (2.0)
Previous medication–no (%)	
Systemic steroids	28 (3.8)
Inhalation steroids	83 (11.3)
Angiotensin converting enzyme inhibitor	126 (17.2)
Angiotensin II receptor blocker	81 (11.0)
Beta–blockers	131 (17.8)
Insulin	46 (6.3)
Metformin	116 (15.8)
Statins	217 (29.6)
Calcium channel blockers	137 (18.7)
Vital signs at day 01	
Heart rate, bpm^**^	84 (74–98)
Mean arterial pressure, mmHg^**^	80 (73–88)
Laboratory tests at day 01	
pH^**^	7.36 (7.30–7.41)
Worst PaO_2_/FiO_2_, mmHg^***^	117 (91–154)
PaCO_2_, mmHg^**^	45 (39–51)
Lactate mmol/L^**^	1.2 (0.9–1.5)
Organ support at day 01–no (%)	
Continuous sedation	703/732 (96.0)
Inotropic or vasopressor	574/732 (78.4)
Vasopressor	573/732 (78.3)
Inotropic	37/732 (5.1)
Fluid balance, mL^****^	637 (77–1445)
Urine output, mL^****^	675 (360–1116)
Ventilation support at day 01	
Assisted ventilation–no (%)^a^	185/731 (25.3)
Volume controlled	125/731 (17.1)
Pressure controlled	421/731 (57.6)
Pressure support	12/731 (1.6)
Synchronized intermittent mandatory ventilation	68/731 (9.3)
Airway pressure release ventilation	3/731 (0.4)
INTELLiVENT-ASV	12/731 (1.6)
Other	90/731 (12.3)
Tidal volume, mL/kg PBW^**^^b^	6.3 (5.9–7.0)
Tidal volume ≤ 8 mL/kg PBW	609 (95.9)
PEEP, cmH_2_O^**^^b^	13 (12–15)
Peak pressure, cmH_2_O^**^^b^	27 (24–30)
Driving pressure, cmH_2_O^**^^b^	14 (12–16)
Driving pressure > 15 cmH_2_O	206/640 (32.2)
Mechanical power, J/min^**^^b^	18.9 (15.7–22.8)
Mechanical power > 17 J/min	381/640 (59.5)
Dynamic compliance, mL/cmH_2_O^**^^b^	32 (27–40)
Total respiratory rate, mpm^**^^b^	22 (20–24)
Set respiratory rate, mpm^**^^b^	22 (20–24)
Minute ventilation, L/min^**^^b^	9.5 (8.4–11.0)
FiO${}_{2}^{**}$	0.60 (0.50–0.70)
etCO_2_, mmHg^**^	37 (32–42)
Rescue therapy at day 01–no (%)	
Prone positioning	225/719 (31.3)
Duration, hours	8 (4–12)
Recruitment maneuver	15/590 (2.5)
ECMO	
Use of NMBA	212/731 (29.0)
Hours of use of use	0 (0–8)
Clinical outcome	
28–day mortality	214 (29.2)

*Data are median (quartile 25%–quartile 75%) or No (%). Percentages may not total 100 because of rounding. CT, computed tomography; PEEP positive end expiratory pressure; ECMO, extracorporeal membrane oxygenation; FiO_2_, inspired fraction of oxygen; PEEP, positive end–expiratory pressure; NMBA, neuromuscular blocking agent.*

* *Most recent measurement in 24 h before intubation, or at ICU admission under invasive ventilation.*

** *Aggregate as the mean of a maximum of four values.*

*** *Worst value of four available.*

**** *Collected in the period after intubation or ICU admission with ventilation until 24:00.*

a *Assisted ventilation defined as any mode other than pressure or volume controlled. The mode of ventilation reported is the mode used 1 h after intubation.*

b *Only assessed in moments without spontaneous breathing activity*.

**Figure 1:**
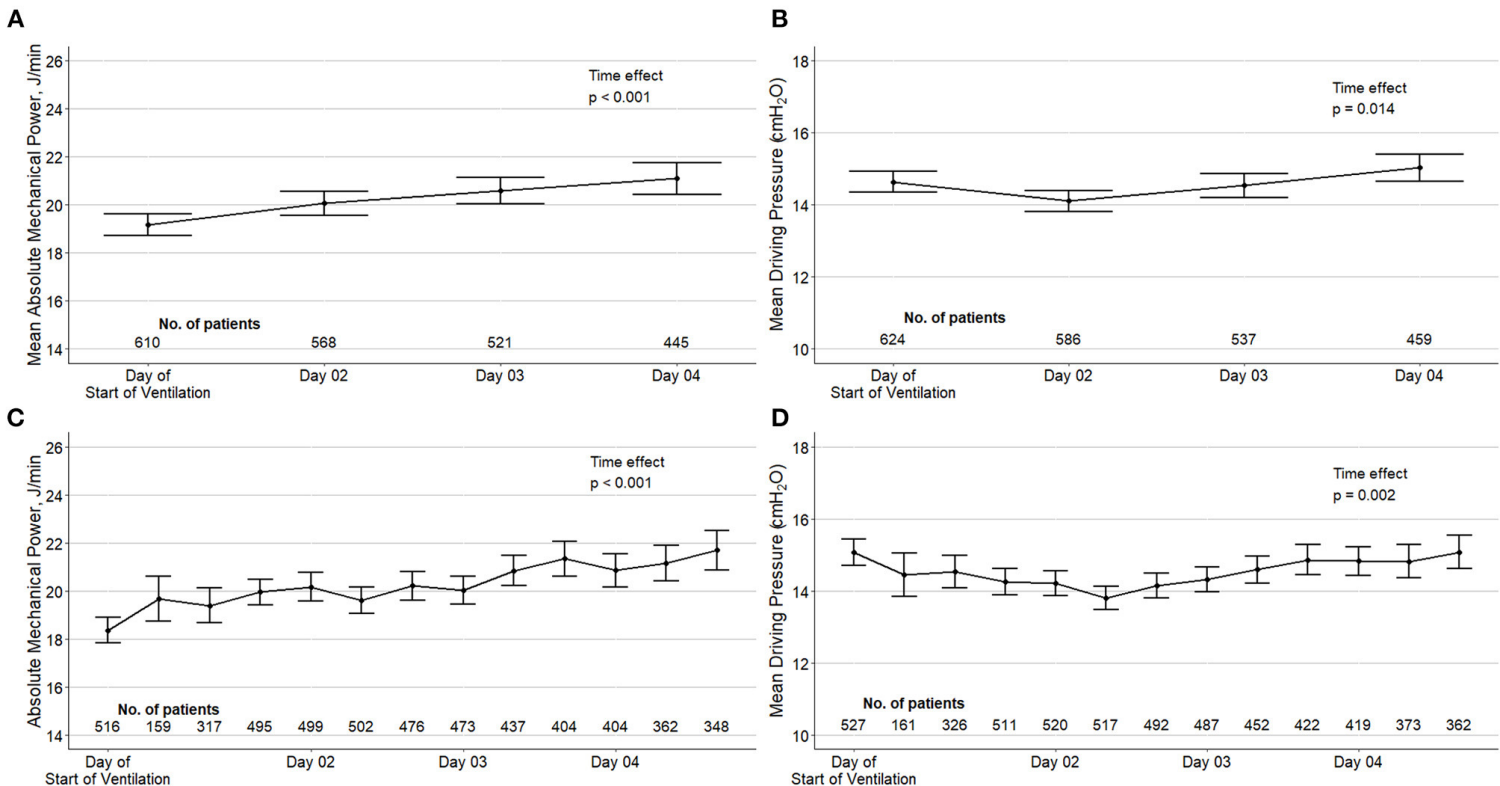
Driving pressure and mechanical power over the first four days of ventilation in the included patients. Top panels **(A, B)**: mean daily values of ΔP and MP according to a maximum of four measurements in the day of start of ventilation and three measurements in the next days. Bottom panels **(C, D)**: ΔP and MP per time point of assessment. Circles are means and error bars are 95% confidence intervals. Both variables were calculated using only measurements without spontaneous breathing activity. *P* values from a mixed-effect model with time as fixed effect (as continuous variable) and patients as random effect to account for repeated measurements.

In the first calendar day of ventilation, median ΔP was 14 (13–17) cm H_2_O, and median MP was 18.9 (15.7–22.8) J/min (Table 1). ΔP was > 15 cm H_2_O in 32.2% of the patients, and MP was > 17 J/min in 59.5% in 66.1% of the patients. ΔP and MP over the first 4 calendar days of ventilation are shown in Figure 1.

**Figure 2:**
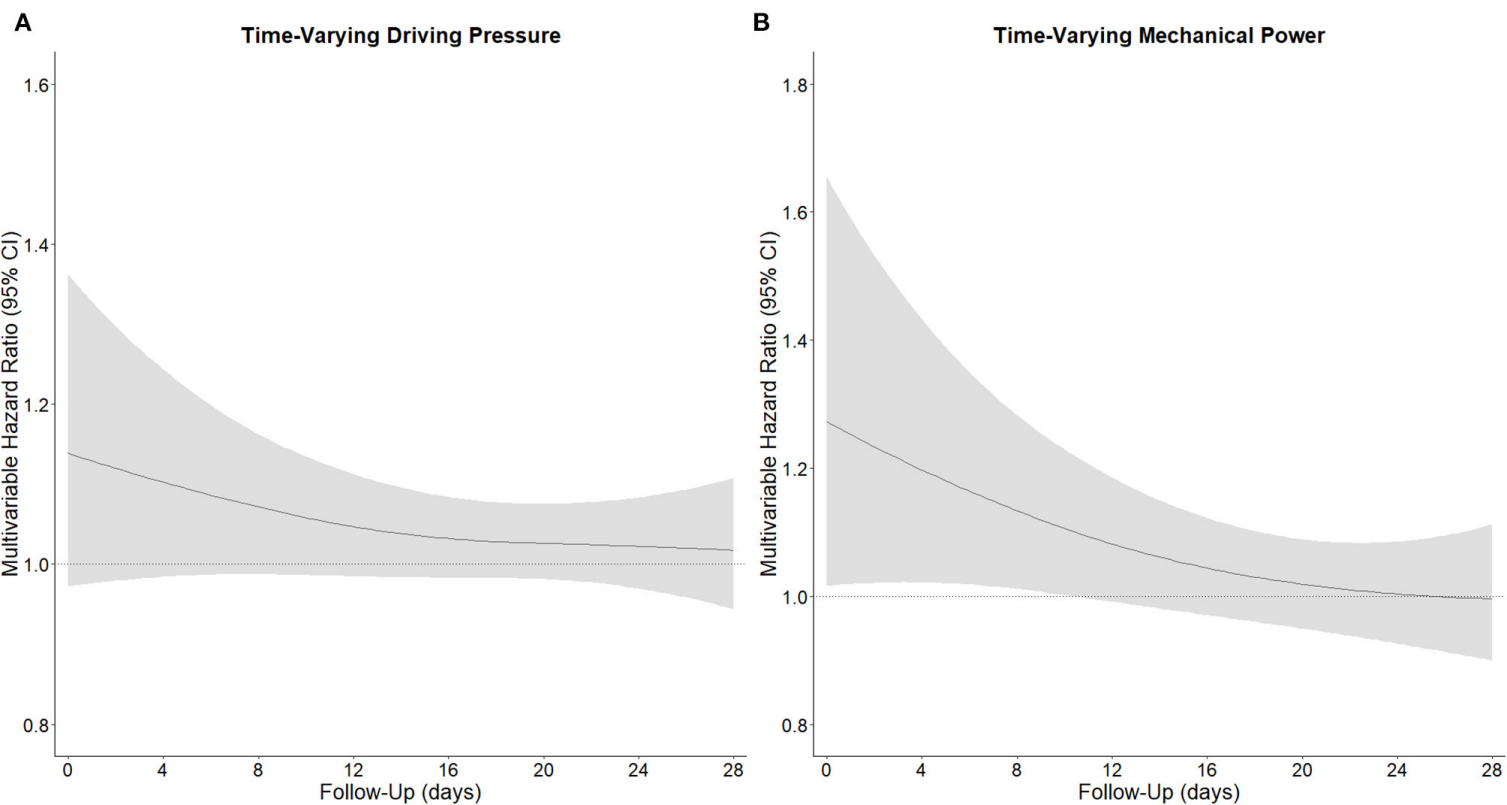
Time-varying hazard ratio of driving pressure and mechanical power for 28-day mortality. **(A)**, Time-varying ΔP, and **(B)** time-varying MP. Time-varying hazard ratio obtained from a Bayesian joint model estimating the association between ΔP and MP and 28-day mortality, including p-splines in an interaction term. The strength of the association decreased over time. All models were adjusted for age, gender, body mass index, PaO_2_ to FiO_2_ ratio, plasma creatinine, medical history of hypertension, heart failure, diabetes, chronic kidney disease, chronic obstructive pulmonary disease, active hematological neoplasia and/or active solid tumor, use of angiotensin converting enzyme inhibitors, use of angiotensin II receptor blockers, use of a vasopressor or inotropes, fluid balance, arterial pH, mean arterial pressure, and heart rate. Natural cubic splines were used in both the fixed-effects and random-effects models to account for the nonlinearity of the longitudinal exposure profiles.

In the first 4 calendar days of ventilation, and after adjusting for confounders, both a high time–varying ΔP (HR, 1.04 [95% CrI, 1.01–1.07]) and a high time–varying MP (HR, 1.12 [95% CrI, 1.01–1.36]) had an association with increased risk of 28–day mortality (Table 2). The strength of the association of ΔP as well as of MP with 28–day mortality decreased slightly over the first 4 days (Figure 2). As shown in the Appendix, the effect of time–varying ΔP was more pronounced in patients with ARDS classified as severe (Supplementary Information Figure 3). The full multivariable model is reported in Supplementary Information Table 3. After adjustment for the daily use of prone positioning and inotropes or vasopressors, both higher ΔP (HR, 1.04 [95% CrI, 1.00–1.09]) and higher MP (HR, 1.06 [95% CrI, 1.00–1.15]) had an association with increased risk of 28–day mortality (Table 2). When restricting the measurements to moment at which neuromuscular blocking agents were administered, both ΔP (HR, 1.38 [95% CrI, 1.14–1.61]) and MP (HR, 1.35 [95% CrI, 1.06–1.73]) had an association with 28–day mortality (Table 2). The findings of the best– and worst–case scenario confirmed the findings of the primary analysis (Table 2).

**Table 2:** Multivariable model assessing the association of time-varying driving pressure and mechanical power over the first four days of ventilation with 28-day mortality.

	**Time-varying models**		**Models of cumulative exposure**	
	**Hazard ratio (95% CrI)**	*p* value^*^	**Hazard ratio (95% CI)**	***p* value**
**Time-varying models (main models)**				
Driving pressure	1.04 (1.01 to 1.07)	0.010	—	—
Mechanical power	1.12 (1.01 to 1.36)	0.018	—	—
**Sensitivity time-varying models**				
Multivariate joint model^**^				
Driving pressure	1.04 (1.00 to 1.09)	0.030	—	—
Mechanical power	1.06 (1.00 to 1.15)	0.049	—	—
Restricted to moments when NMBA was used				
Driving pressure	1.38 (1.14 to 1.61)	<0.001	—	—
Mechanical power	1.35 (1.06 to 1.73)	<0.001	—	—
Best-case scenario^***^				
Driving pressure	1.03 (1.01 to 1.07)	0.007		
Mechanical power	1.13 (1.02 to 1.32)	0.014		
Worst-case scenario^***^				
Driving pressure	1.03 (1.00 to 1.07)	0.018		
Mechanical power	1.09 (1.01 to 1.22)	0.014		
**Models of cumulative exposure**				
Percentage of moments with ΔP > 15 cmH_2_O	—	—	1.61 (1.03 to 2.51)	0.037
Percentage of moments with MP > 17 J/min	—	—	2.42 (1.44 to 4.07)	<0.001
Cumulative dose of ΔP > 15 cmH_2_O	—	—	1.09 (1.05 to 1.14)	<0.001
Cumulative dose of MP > 17 J/min	—	—	1.06 (1.03 to 1.09)	<0.001
Time-weighted average ΔP	—	—	1.06 (1.03 to 1.09)	<0.001
Time-weighted average MP	—	—	1.04 (1.02 to 1.06)	<0.001

*CrI, credible interval; CI, confidence interval; NMBA, neuromuscular blocking agents. Hazard ratios were the adjusted hazard ratios associated with a 1-standard deviation increment. Values higher than 1 indicate increased mortality.*

* *P values calculated as the tail probabilities using the formula 2 x min {P(θ > 0), P(θ <0)}, with θ denoting the corresponding regression coefficient from the survival submodel.*

** *Expansion of the original joint models further adjusted by the following time-varying covariates: daily use of prone positioning and daily use of inotropes or vasopressor.*

*** *In the best-case scenario, 20 patients with missing in 28-day mortality were considered alive at day 28. In the worst-case scenario, 20 patients with missing in 28-day mortality were considered dead at day 28*.

**Figure 3:**
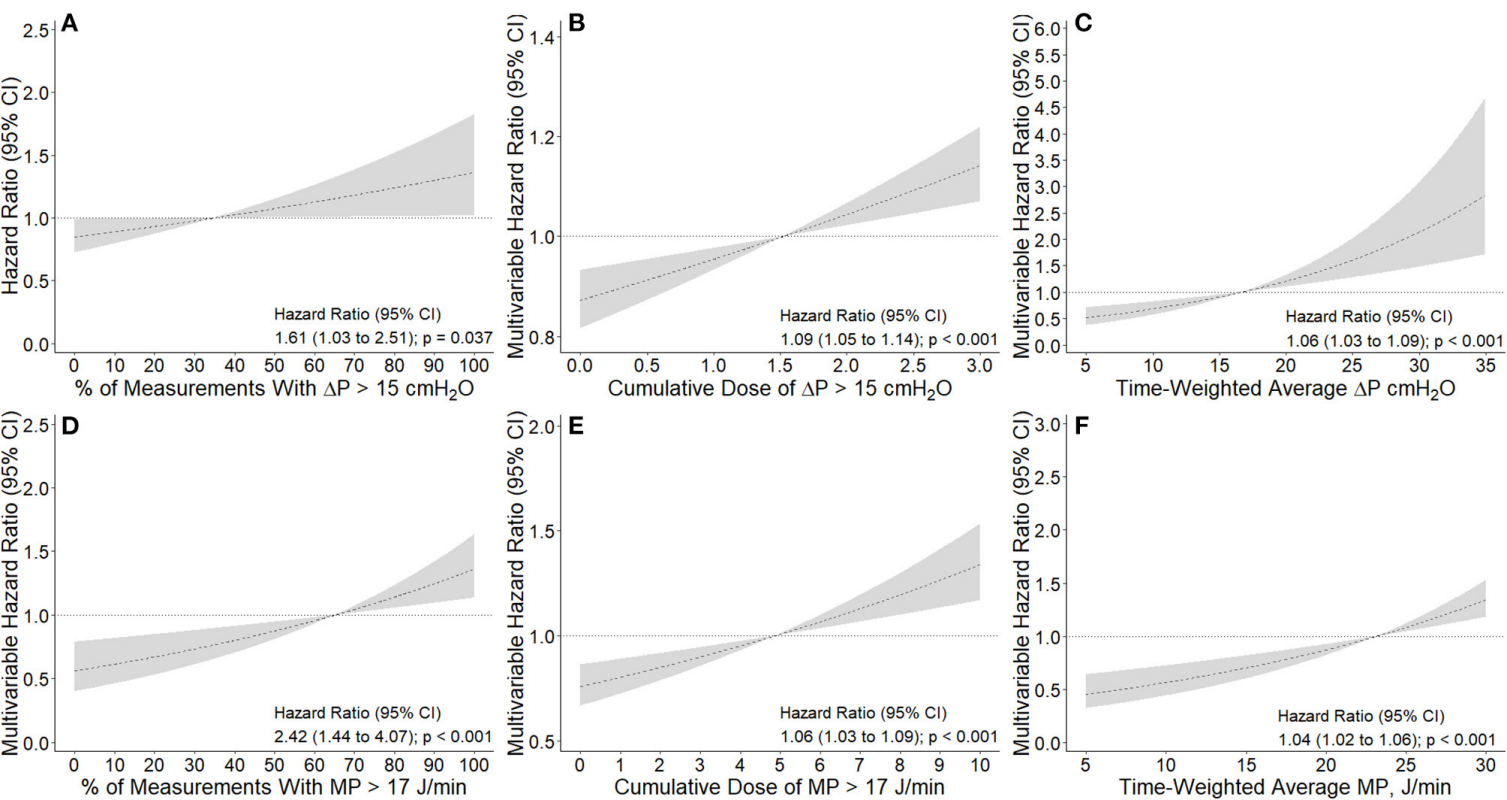
Association between intensity of exposure to higher driving pressure and mechanical power and 28-day mortality. **(A, D):** Association between percentage of measurements with ΔP > 15 cmH_2_O and MP > 17 J/min and 28-day mortality. Percentage calculated from a maximum of 13 measurements extracted every 8 h. **(B, E):** Association between cumulative dose of ΔP > 15 cmH_2_O and MP > 17 J/min and 28-day mortality. Cumulative dose calculated as area under the ΔP and MP time curve above the thresholds described above divided by the number of hours of exposure, as a measure of dose. Using this definition, 1 cmH_2_O or 1 J/min of dose describes that a patient's average ΔP or MP was 1 cmH_2_O or 1 J/min above the thresholds described for the duration of the exposure window, respectively. **(C,F)** Association between time-weighted average ΔP and MP and 28-day mortality. Time-weighted average calculated as the area under the ΔP and MP time curve divided by the number of hours of exposure. All models were adjusted for age, gender, body mass index, PaO_2_ to FiO_2_ ratio, plasma creatinine, medical history of hypertension, heart failure, diabetes, chronic kidney disease, chronic obstructive pulmonary disease, active hematological neoplasia and/or active solid tumor, use of angiotensin converting enzyme inhibitors, use of angiotensin II receptor blockers, use of a vasopressor or inotropes, fluid balance, arterial pH, mean arterial pressure, and heart rate. Dashed lines and gray areas represent hazard ratio and 95% confidence interval for increasing values the variable analyzed as a continuous variable and centralized in the mean of each variable. Δ*P is driving pressure and MP is mechanical power*.

The number and percentage of measurements per patient with ΔP > 15 cm H_2_O and MP > 17 J/min in the first 4 days of ventilation are shown in the Appendix (Supplementary Information Figure 4). A higher percentage of measurements with ΔP > 15 cm H_2_O (HR, 1.61 [95% CI, 1.03–2.51] *p* = 0.037) or with MP > 17 J/min (HR, 2.42 [95% CI, 1.44–4.07] *p* < 0.001) had an association with increased risk of 28–day mortality (Figure 3). Comparison of baseline characteristics and outcomes according to the median ΔP and MP in the first 4 calendar days is shown in Supplementary Information Table 4.

Time–weighted average and cumulative dose of ΔP and MP are shown in the Appendix (Supplementary Information Figure 5). Cumulative dose of ΔP > 15 cm H_2_O (HR, 1.09 [95% CI, 1.05–1.14]; *p* < 0.001) and MP > 17 J/min (HR, 1.06 [1.03–1.09]; *p* < 0.001) had an association with increased risk of 28–day mortality (Figure 3). In accordance, time–weighted average ΔP (HR, 1.06 [95% CI, 1.03–1.09]; *p* < 0.001) and time–weighted average MP (HR, 1.04 [95% CI, 1.02–1.06]; *p* < 0.001) also had an association with an increased risk on 28–day mortality.

## Discussion

The findings of this observational study assessing the impact of time–varying ΔP and MP on 28–day mortality in invasively ventilated COVID−19 patients with ARDS, can be summarized as follows: (1) exposure to both higher ΔP and higher MP during the first 4 calendar days of ventilation had associations with increased risk of 28–day mortality in joint models; and (2) a higher cumulative exposure to ΔP > 15 cm H_2_O or MP > 17 J/min over the first 4 days of ventilation had associations with increased risk for 28–day mortality. These findings support the suggestion that limiting the intensity of ventilation in COVID−19 patients with ARDS by using strategies that result in a lower ΔP or MP could improve patient outcome, alike what has been suggested in patients with ARDS from another origin.

The findings of our study are in line with previous investigations of ΔP and MP in patients with ARDS from another cause than COVID−19. Indeed, in patients with ARDS from another origin, a higher ΔP and higher MP at baseline have been shown to have an association with increased mortality (7–10, 12–16). A recent study showed the adverse effect of exposure to higher cumulative doses of ΔP and MP over the duration of ventilation in critically ill patients receiving ventilation due to respiratory failure (12). We also found that a high time–varying ΔP and MP, and a higher cumulative exposure to harmful levels of ΔP and MP have an association with increased 28–day mortality, and as in the previous study these effects were more pronounced in patients with more severe forms of ARDS at baseline.

Considering all the available evidence, monitoring intensity of ventilation seems an attractive approach, both in patients with ARDS due to COVID−19 and in patients with ARDS from another origin. Yet, it remains uncertain whether the found association only reflects the relationship between respiratory system compliance and patient outcome. It is also uncertain whether measures to lower the intensity will result in better outcomes. Nevertheless, our results, corrected for baseline covariates and time–varying confounding, could represent a causal effect. Thus, ventilation strategies aiming to lower the intensity of ventilation need to be studied, preferably in randomized clinical trials.

The present cohort of COVID−19 patients with ARDS is comparable to other COVID−19 cohorts in relation to baseline characteristics and clinical outcomes (28–31). Key variables of ventilation management, like V_T_, RR and ΔP were comparable to what was described in previous studies in patients with COVID−19, and in line with recommendations for lung–protective ventilation in patients with ARDS (28–31). MP in our cohort was lower than what was reported in one study in COVID−19 patients, (30) suggesting a better adoption of lung–protective ventilation in our study.

Our study has strengths. We restricted the analysis to patients who were without spontaneous breathing activity for the majority of time, and ΔP and MP was only calculated for the moments a patient was passive. Calculation ΔP and MP in the presence of spontaneous breathing is not yet validated. For the main analyses we used joint models, which allows the examination of the effect of a time–varying, endogenous covariate on a time–to–event outcome, accounting for non–random dropouts due to death during follow–up.

Our study also has limitations. First, our study was conducted very early in the local outbreak, and anti–inflammatory strategies, like steroids and other treatments such as anti–IL−6, were not yet extensively used. We did not collect data regarding the use of steroids or other anti–inflammatory drugs. Also, we did not collect D–dimer levels and other laboratory tests that may have predictive value, as these were not yet routinely performed so early in the local outbreak. However, the variables used for adjustment are in line with previous studies assessing the impact of ΔP and MP in patients receiving ventilation (12, 14, 15). Our models used several important clinical variables. Of note, we only had measurements of dynamic airway ΔP as plateau or transpulmonary pressures were not routinely connected. Airway ΔP does correlate with transpulmonary ΔP, but it represents a surrogate, which might be affected by other factors. However, recent data suggested that this calculation is reliable (32). Also, we could not adjust for all possible yet unmeasured confounders and due to the observational nature, no causal relationship can be inferred or determined. A high incidence of thromboembolic complications has been found in the present study, which is in line with other reports on COVID−19 patients (33). Pulmonary embolism could cause an increase in dead space, as such affecting the intensity of ventilation. Another limitation is that participating centers did not all use the same disease severity score. Indeed, some centers reported either APACHE II or APACHE IV scores, others used the SAPS II, and some only SOFA scores. These scores cannot be used interchangeably. However, several baseline covariates were used in our models, representing all organ systems, several supportive treatments and pre–existing comorbidities. The mortality rate in our study is lower than that reported in one recent cohort of COVID−19 patients, (2) but higher than in other reports (34, 35). It remains to be determined whether the associations found here are also present in cohorts with other mortality rates. In addition, the models used had some limitations. All baseline covariates were assumed to be measured without error. The joint models assume the correct specification of random effect structure, and all interdependencies between longitudinal and time–to–event outcomes should be explained by the latent, subject–specific random effects structure. Also, there is a risk of residual confounding.

## Conclusions

In conclusion, in this cohort of COVID−19 patients with ARDS, exposure to higher ΔP or MP, or to a higher cumulative exposure to a ΔP > 15 cm H_2_O or a MP > 17 J/min over the first 4 calendar days of ventilation had associations with increased risk for 28–day mortality. To ascertain a causal relationship between ΔP and MP with mortality, randomized clinical trials are needed.

## Data Availability Statement

The raw data supporting the conclusions of this article will be made available by the authors, without undue reservation.

## Ethics Statement

The studies involving human participants were reviewed and approved by the institutional review boards of each participating center. Written informed consent for participation was not required for this study in accordance with the national legislation and the institutional requirements.

## PRoVENT–COVID Collaborative Group

Monitored email address: provent-covid@amsterdamumc.nl.

**Investigators:** J.P. van Akkeren; A.G. Algera; C.K. Algoe; R.B. van Amstel; O.L. Baur; P. van de Berg; D.C.J.J. Bergmans; D.I. van den Bersselaar; F.A. Bertens; A.J.G.H. Bindels; M.M. de Boer; S. den Boer; L.S. Boers; M. Bogerd; L.D.J. Bos; M. Botta; J.S. Breel; H. de Bruin; S. de Bruin; C.L. Bruna; L.A. Buiteman–Kruizinga; O. Cremer; R.M. Determann; W. Dieperink; D.A. Dongelmans; H.S. Franke; M.S. Galek Aldridge; M.J. de Graaff; L.A. Hagens; J.J. Haringman; N.F.L. Heijnen; S.Hiel; S.T. van der Heide; P.L.J. van der Heiden; L.L. Hoeijmakers; L. Hol; M. W. Hollmann; M.E. Hoogendoorn; J. Horn; R. van der Horst; E.L.K. Ie; D. Ivanov; N.P. Juffermans; E. Kho; E.S. de Klerk; A.W.M. Koopman; M. Koopmans; S. Kucukcelebi; M.A. Kuiper; D.W. de Lange; D.M.P. van Meenen; I. Martin–Loeches, G. Mazzinari; N. van Mourik; S.G. Nijbroek; M. Onrust; E.A.N. Oostdijk; F. Paulus; C.J. Pennartz; J. Pillay; L. Pisani; I.M. Purmer; T.C.D. Rettig; J.P Roozeman; M.T.U. Schuijt; M.J. Schultz; A. Serpa Neto; M.E. Sleeswijk; M.R. Smit; P.E. Spronk; W. Stilma; A.C. Strang; A.M. Tsonas; P.R Tuinman; C.M.A. Valk; F.L. Veen; A.P.J. Vlaar; L.I. Veldhuis; P. van Velzen; W.H. van der Ven; P. van Vliet; P. van der Voort; H.H. van der Wier; L. van Welie; H.J.F.T. Wesselink; B. van Wijk; T. Winters; W.Y. Wong; A.R.H. van Zanten.

## Author Contributions

MiS and AS had full access to all data in the study and take responsibility for the integrity of the data and the accuracy of the data analysis. MiS, MaS, GM, FP, and AS concept and design. All authors contributed to acquisition, analysis or interpretation of data, drafting the manuscript, and critical revision of the manuscript for important intellectual content. MiS and AS statistical analysis. FP and MaS obtained funding. MaS, FP, and AS supervision. All authors contributed to acquisition, analysis or interpretation of data, drafting the manuscript, and critical revision of the manuscript for important intellectual content. All authors contributed to the article and approved the submitted version.

## Funding

The Amsterdam University Medical Centers, location AMC funded this study. It had no role in the design, analysis, interpretation of data, writing or submission of this study.

## Conflict of Interest

AS reports personal fees from Dräger, outside of the submitted work. MaS reports personal fees from Hamilton and Xenios/Novalung, outside of the submitted work. The remaining authors declare that the research was conducted in the absence of any commercial or financial relationships that could be construed as a potential conflict of interest.

## Publisher's Note

All claims expressed in this article are solely those of the authors and do not necessarily represent those of their affiliated organizations, or those of the publisher, the editors and the reviewers. Any product that may be evaluated in this article, or claim that may be made by its manufacturer, is not guaranteed or endorsed by the publisher.
