# Supplementary material for: Association of Time–Varying Intensity of Ventilation With Mortality in Patients With COVID−19 ARDS: Secondary Analysis of the PRoVENT–COVID Study
Source: Front Med (Lausanne). 2021 Nov 18;8:725265. doi: 10.3389/fmed.2021.725265 (PMC8637438; doi:10.3389/fmed.2021.725265)
Supplement: Supplementary file 1 [file Data_Sheet_1.PDF]

Association of Time–Varying Intensity of Ventilation with Mortality in Patients with COVID–19 ARDS: Secondary analysis of the PRoVENT–COVID study

ONLINE SUPPLEMENT

eMethods

Data preparation and selection of the cohort

At each time point, presence of spontaneous breathing was likely if: 1) patient was on a spontaneous ventilation mode, e.g., pressure support ventilation; or 2) patient was on a non–spontaneous ventilation mode with measured (total) respiratory rate exceeding the set respiratory rate > 2 breaths per minute. For the final assessment, only patients with 50% or more of the measurements available in the first four days of ventilation without evidence of spontaneous breathing were included.

Rationale for selection of the cohort

In the present study, the cohort of patients was restricted–patients receiving mandatory ventilation for the majority of the time, and the calculation of driving pressure and mechanical power was restricted–moments without evidence of spontaneous breathing. Indeed, the calculation of driving pressure and mechanical power in the presence of spontaneous breathing is not yet validated. In assisted modes of ventilation, the derivation of driving pressure and mechanical power is challenging using established methods as airway pressure, flow and pleural pressure are affected counter-directionally and simultaneously overlapping by the action of the ventilator and the respiratory muscles [1]. Even if appropriately adjusted for resistance, flow, and chest wall elastance, any estimate of these variables during spontaneous efforts would reflect both the ventilator's contribution and respiratory muscle activity, and thus would not represent the total energy imparted during inflation [2].

Imputation performance

When driving pressure and mechanical power were missing in moments classified as without spontaneous breathing, a linear imputation method was used–impute values with a limit of 16 hours between each measurement available. When the gap between available measurements exceeded 16 hours, the value was considered missing.

Over the first four days, 5569 measurements of driving pressure were available before imputation, and 5629 after, representing a median of 9 (5 – 11) measurements per patient, before and after imputation. In relation–mechanical power, 5392 measurements before, and 5470 measurements after imputation were available, representing a median of 8 (4 – 11) measurements per patient before imputation, and 9 (4 – 11) after imputation. The distribution of the variables before and after linear imputation was similar (**eFigure 1**).

References

1. Huhle R, Serpa Neto A, Schultz MJ, Gama de Abreu M. Is mechanical power the final word on ventilator-induced lung injury?—no. *Ann Transl Med*. 2018;6(19):394. doi:10.21037/atm.2018.09.65
2. Cressoni M, Cadringher P, Chiurazzi C, et al. Lung inhomogeneity in patients with acute respiratory distress syndrome. *Am J Respir Crit Care Med*. 2014;189(2):149-158. doi:10.1164/rccm.201308-1567OC

eTable 1–Rate of Missing Data

	**Original Cohort (*n* = 734)**
Age	0 (0.0)
Gender	0 (0.0)
Body mass index	7 (1.0)
Severity of ARDS	10 (1.4)
Hypertension	0 (0.0)
Heart failure	0 (0.0)
Diabetes	0 (0.0)
Chronic kidney disease	0 (0.0)
Baseline creatinine	20 (2.7)
Liver cirrhosis	0 (0.0)
Chronic obstructive pulmonary disease	0 (0.0)
Active hematological neoplasia	0 (0.0)
Active solid neoplasia	0 (0.0)
Neuromuscular disease	0 (0.0)
Immunosuppression	0 (0.0)
Systemic steroids	0 (0.0)
Inhalation steroids	0 (0.0)
Angiotensin converting enzyme inhibitor	0 (0.0)
Angiotensin II receptor blocker	0 (0.0)
Beta-blockers	0 (0.0)
Insulin	0 (0.0)
Metformin	0 (0.0)
Statins	0 (0.0)
Calcium channel blockers	0 (0.0)
Heart rate	1 (0.1)
Mean arterial pressure	4 (0.5)
pH	6 (0.8)
PaO_2 / FiO_2	0 (0.0)
$PaCO_2$	6 (0.8)
Lactate	97 (13.2)
Use of continuous sedation	2 (0.3)
Use of vasopressor	2 (0.3)
Use of inotropic drugs	2 (0.3)
Fluid balance	15 (2.0)
Urine output	18 (2.5)
Prone positioning	15 (2.0)
Recruitment maneuver	144 (19.6)
Extracorporeal membrane oxygenation	13 (1.8)

eTable 1–Rate of Missing Data

	**Original Cohort (*n* = 734)**
Use of neuromuscular blocking agent	3 (0.4)
28-Day mortality	0 (0.0)

eTable 2–Additional Clinical Outcomes in the Included Cohort

	**Original Cohort (*n* = 734)**
Ventilator-free days at day 28, days	0.0 (0.0–15.0)
Duration of ventilation, days	15.0 (9.0–24.0)
In survivors at day 28, days	17.0 (11.0–29.0)
Tracheostomy – no (%)	128 (17.6)
Thromboembolic complications – no (%)	217 (29.6)
Pulmonary embolism	176 (24.0)
Deep vein thrombosis	30 (4.1)
Ischemic stroke	23 (3.1)
Myocardial infarction	8 (1.1)
Systemic arterial embolism	3 (0.4)
Acute kidney injury – no (%)	350 (47.9)
Need for RRT – no (%)	151 (20.6)
Need of rescue therapy – no (%)*	567 (77.8)
Prone positioning	437 (59.9)
Recruitment maneuver	41 (6.9)
Use of NMBA	382 (52.0)
ECMO	9 (1.2)
ICU length of stay, days	16.0 (10.0–28.0)
In survivors, days	20.0 (12.0–31.0)
Hospital length of stay, days	25.0 (14.0–38.0)
In survivors, days	31.0 (21.0–45.0)
ICU mortality – no (%)	244 (33.7)
Hospital mortality – no (%)	249 (36.6)
90-day mortality – no (%)	258 (37.6)

Data are median (quartile 25%–quartile 75%) or No (%). Percentages may not total 100 because of rounding

RRT: renal replacement therapy; NMBA: neuromuscular blocking agent; ECMO: extracorporeal membrane oxygenation; ICU: intensive care unit

* assessed in the first four days of ventilation

eTable 3–Additional Variables Included in the Multivariable Model

	Time-Varying Model for ΔP		Time-Varying Model for MP	
	**Hazard Ratio (95% CrI)**	***p* value**	**Hazard Ratio (95% CrI)**	***p* value**
**Demographic characteristics**				
Age	1.88 (1.55 to 2.31)	< 0.001	2.03 (1.64 to 2.62)	< 0.001
Male gender	1.16 (0.81 to 1.69)	0.439	1.08 (0.70 to 1.68)	0.753
Body mass index	0.93 (0.78 to 1.11)	0.478	0.93 (0.76 to 1.13)	0.435
**Co-existing disorders**				
Hypertension	1.38 (0.93 to 2.02)	0.103	1.45 (1.01 to 2.16)	0.046
Heart failure	1.10 (0.52 to 2.06)	0.707	1.19 (0.56 to 2.33)	0.642
Diabetes	1.24 (0.89 to 1.73)	0.225	1.22 (0.79 to 1.77)	0.314
Chronic kidney disease	0.73 (0.37 to 1.50)	0.392	0.82 (0.37 to 1.61)	0.610
Chronic obstructive pulmonary disease	2.19 (1.46 to 3.34)	< 0.001	2.28 (1.47 to 3.69)	< 0.001
Hematological neoplasia	1.45 (0.35 to 4.44)	0.489	1.97 (0.45 to 6.75)	0.317
Solid tumor	1.12 (0.46 to 2.48)	0.714	1.09 (0.40 to 2.49)	0.807
**Previous medication**				
Angiotensin converting enzyme inhibitor	0.87 (0.56 to 1.35)	0.550	0.78 (0.52 to 1.17)	0.296
Angiotensin II receptor blocker	0.82 (0.50 to 1.32)	0.457	0.85 (0.50 to 1.42)	0.557
**Organ support at day 01**				
Use of inotropic or vasopressor	1.13 (0.75 to 1.72)	0.528	1.13 (0.73 to 1.85)	0.642
Fluid balance	1.03 (0.88 to 1.20)	0.600	1.07 (0.90 to 1.26)	0.460
**Laboratory tests at day 01**				
PaO_2 / FiO_2	0.87 (0.72 to 1.04)	0.139	0.84 (0.65 to .03)	0.082
Creatinine	1.13 (0.98 to 1.27)	0.067	1.14 (0.98 to 1.29)	0.071
pH	0.76 (0.65 to 0.91)	0.003	0.75 (0.61 to 0.92)	0.003
**Vital signs at day 01**				
Mean arterial pressure	0.83 (0.70 to 0.97)	0.021	0.85 (0.70 to 1.01)	0.085
Heart rate	1.27 (1.09 to 1.52)	0.003	1.32 (1.09 to 1.62)	0.003

CrI: credible interval

For continuous variables hazard ratios were the adjusted hazard ratios associated with a 1-standard deviation increment. Values higher than 1 indicate increased mortality.

* *P* values calculated as the tail probabilities using the formula 2 x min{P($\theta > 0$), P($\theta < 0$)}, with θ denoting the corresponding regression coefficient from the survival submodel.

eTable 4 – Baseline patient characteristics and outcomes according to the median driving pressure or mechanical power in the first four days

	**ΔP > 15 cmH_2O (*n* = 245)**	**ΔP ≤ 15 cmH_2O (*n* = 420)**	***p* value**	**MP > 17 J/min (*n* = 443)**	**MP ≤ 17 J/min (*n* = 207)**	***p* value**
Age, years	63.0 (55.0 - 71.0)	65.5 (57.0 - 72.0)	0.077	64.0 (56.0 - 71.0)	66.0 (58.5 - 72.0)	0.073
Male gender – no (%)	165 (67.3)	320 (76.2)	0.015	359 (81.0)	114 (55.1)	< 0.001
Body mass index, kg/m^2	28.6 (25.9 - 32.8)	27.5 (25.1 - 30.1)	< 0.001	28.1 (25.9 - 31.8)	27.2 (25.0 - 30.1)	0.002
Transferred under invasive ventilation	27 (11.0)	65 (15.5)	0.130	70 (15.8)	22 (10.6)	0.091
Days between intubation and admission	0.0 (0.0 - 0.0)	0.0 (0.0 - 0.0)	0.760	0.0 (0.0 - 0.0)	0.0 (0.0 - 0.0)	0.077
Use of non–invasive ventilation prior to intubation – no (%)	17 (7.5)	42 (11.1)	0.160	44 (10.9)	15 (8.0)	0.305
Duration of non–invasive ventilation, hours	6.5 (2.0 - 16.5)	4.0 (2.0 - 12.5)	0.624	4.8 (2.0 - 8.9)	9.5 (2.5 - 22.6)	0.232
Chest CT scan performed – no (%)	99 (41.8)	109 (26.5)	< 0.001	148 (34.5)	53 (25.9)	0.029
Lung parenchyma affected – no (%)			0.067			0.009
0%	0 (0.0)	8 (7.4)		2 (1.4)	6 (11.3)	
25%	30 (30.3)	30 (27.8)		39 (26.5)	20 (37.7)	
50%	35 (35.4)	32 (29.6)		49 (33.3)	15 (28.3)	
75%	27 (27.3)	29 (26.9)		44 (29.9)	9 (17.0)	
100%	7 (7.1)	9 (8.3)		13 (8.8)	3 (5.7)	
Chest X–ray performed – no (%)	113 (80.7)	270 (91.2)	0.003	240 (86.3)	135 (90.0)	0.287
Quadrants affected – no (%)			0.052			0.320
1	3 (2.6)	25 (9.4)		15 (6.3)	13 (9.8)	
2	32 (28.1)	64 (24.1)		56 (23.4)	36 (27.1)	
3	30 (26.3)	83 (31.2)		71 (29.7)	41 (30.8)	
4	49 (43.0)	94 (35.3)		97 (40.6)	43 (32.3)	
Severity of ARDS – no (%)			0.003			0.037

eTable 4 – Baseline patient characteristics and outcomes according to the median driving pressure or mechanical power in the first four days

	**ΔP > 15 cmH_2O (*n* = 245)**	**ΔP ≤ 15 cmH_2O (*n* = 420)**	***p* value**	**MP > 17 J/min (*n* = 443)**	**MP ≤ 17 J/min (*n* = 207)**	***p* value**
Mild	15 (6.1)	44 (10.5)		31 (7.0)	27 (13.0)	
Moderate	130 (53.1)	255 (60.7)		269 (60.7)	112 (54.1)	
Severe	100 (40.8)	121 (28.8)		143 (32.3)	68 (32.9)	
Co–existing disorders – no (%)						
Hypertension	78 (31.8)	145 (34.5)	0.497	148 (33.4)	71 (34.3)	0.859
Heart failure	14 (5.7)	12 (2.9)	0.095	19 (4.3)	7 (3.4)	0.672
Diabetes	55 (22.4)	92 (21.9)	0.923	107 (24.2)	38 (18.4)	0.106
Chronic kidney disease	5 (2.0)	23 (5.5)	0.044	16 (3.6)	11 (5.3)	0.301
Baseline creatinine, µmol/L*	80.0 (61.8 - 99.0)	77.0 (62.0 - 100.0)	0.690	80.5 (65.0 - 102.0)	69.0 (54.0 - 90.2)	< 0.001
Liver cirrhosis	1 (0.4)	1 (0.2)	0.999	1 (0.2)	1 (0.5)	0.536
Chronic obstructive pulmonary disease	21 (8.6)	36 (8.6)	0.999	42 (9.5)	13 (6.3)	0.226
Active hematological neoplasia	1 (0.4)	8 (1.9)	0.165	5 (1.1)	3 (1.4)	0.714
Active solid neoplasia	5 (2.0)	13 (3.1)	0.470	10 (2.3)	8 (3.9)	0.304
Neuromuscular disease	1 (0.4)	1 (0.2)	0.999	2 (0.5)	0 (0.0)	0.999
Immunosuppression	7 (2.9)	7 (1.7)	0.401	9 (2.0)	5 (2.4)	0.775
Previous medication – no (%)						
Systemic steroids	10 (4.1)	16 (3.8)	0.839	14 (3.2)	10 (4.8)	0.371
Inhalation steroids	35 (14.3)	41 (9.8)	0.100	53 (12.0)	21 (10.1)	0.596
Angiotensin converting enzyme inhibitor	32 (13.1)	79 (18.8)	0.067	72 (16.3)	38 (18.4)	0.503
Angiotensin II receptor blocker	25 (10.2)	46 (11.0)	0.796	49 (11.1)	21 (10.1)	0.787
Beta–blockers	49 (20.0)	67 (16.0)	0.204	74 (16.7)	39 (18.8)	0.507

eTable 4 – Baseline patient characteristics and outcomes according to the median driving pressure or mechanical power in the first four days

	ΔP > 15 cmH_2O (*n* = 245)	ΔP ≤ 15 cmH_2O (*n* = 420)	*p* value	MP > 17 J/min (*n* = 443)	MP ≤ 17 J/min (*n* = 207)	*p* value
Insulin	15 (6.1)	27 (6.4)	0.999	28 (6.3)	12 (5.8)	0.863
Metformin	40 (16.3)	66 (15.7)	0.827	77 (17.4)	28 (13.5)	0.253
Statins	69 (28.2)	124 (29.5)	0.724	137 (30.9)	53 (25.6)	0.195
Calcium channel blockers	46 (18.8)	84 (20.0)	0.761	77 (17.4)	51 (24.6)	0.034
Vital signs at day 01						
Heart rate, bpm**	88.0 (76.5 - 101.5)	84.0 (74.6 - 95.8)	0.004	87.3 (75.7 - 100.3)	81.0 (72.8 - 92.2)	< 0.001
Mean arterial pressure, mmHg**	79.5 (73.2 - 88.5)	80.5 (73.9 - 87.7)	0.778	79.5 (73.3 - 87.9)	80.0 (75.0 - 87.7)	0.534
Laboratory tests at day 01						
pH**	7.35 (7.30 - 7.40)	7.36 (7.31 - 7.41)	0.007	7.35 (7.30 - 7.40)	7.38 (7.32 - 7.42)	< 0.001
Worst PaO_2 / FiO_2, mmHg***	110.8 (87.2 - 142.5)	125.5 (94.7 - 164.8)	0.001	121.4 (92.4 - 155.5)	116.1 (87.0 - 150.7)	0.387
$PaCO_2$, mmHg**	46.5 (41.3 - 54.1)	43.9 (39.0 - 49.5)	< 0.001	45.5 (40.5 - 52.5)	43.1 (37.8 - 49.0)	< 0.001
Lactate mmol/L**	1.2 (0.9 - 1.6)	1.1 (0.9 - 1.4)	0.002	1.2 (0.9 - 1.5)	1.0 (0.8 - 1.2)	< 0.001
Organ support at day 01 – no (%)						
Continuous sedation	235 (95.9)	400 (95.7)	0.999	429 (97.1)	192 (93.2)	0.033
Inotropic or vasopressor	191 (78.0)	324 (77.5)	0.923	349 (79.0)	156 (75.7)	0.361
Vasopressor	190 (77.6)	324 (77.5)	0.999	348 (78.7)	156 (75.7)	0.417
Inotropic	19 (7.8)	17 (4.1)	0.051	30 (6.8)	6 (2.9)	0.045
Fluid balance, mL****	644 (81 - 1463)	605 (54 - 1443)	0.936	660 (65 - 1468)	567 (60 - 1344)	0.497
Urine output, mL****	692 (387 - 1225)	675 (350 - 1107)	0.178	675 (380 - 1158)	650 (350 - 1097)	0.234
Ventilation support at day 01						
Assisted ventilation – no (%)[a]	46 (18.8)	78 (18.7)	0.999	95 (21.5)	28 (13.6)	0.018

eTable 4 – Baseline patient characteristics and outcomes according to the median driving pressure or mechanical power in the first four days

	ΔP > 15 cmH_2O (*n* = 245)	ΔP ≤ 15 cmH_2O (*n* = 420)	*p* value	MP > 17 J/min (*n* = 443)	MP ≤ 17 J/min (*n* = 207)	*p* value
Volume controlled	65 (26.5)	60 (14.4)		95 (21.5)	30 (14.6)	
Pressure controlled	134 (54.7)	279 (66.9)		252 (57.0)	148 (71.8)	
Pressure support	4 (1.6)	8 (1.9)		10 (2.3)	1 (0.5)	
Synchronized intermittent mandatory ventilation	26 (10.6)	42 (10.1)		51 (11.5)	17 (8.3)	
Airway pressure release ventilation	2 (0.8)	1 (0.2)		2 (0.5)	1 (0.5)	
INTELLiVENT-ASV	3 (1.2)	9 (2.2)		7 (1.6)	5 (2.4)	
Other	11 (4.5)	18 (4.3)		25 (5.7)	4 (1.9)	
Tidal volume, mL/kg PBW**[,b]	6.4 (5.9 - 7.1)	6.3 (5.9 - 6.9)	0.200	6.3 (5.9 - 7.0)	6.3 (5.8 - 7.0)	0.410
Tidal volume ≤ 8 mL/kg PBW	223 (96.1)	386 (95.8)	0.999	416 (96.3)	193 (95.1)	0.521
PEEP, cmH_2O**[,b]	13.3 (11.5 - 15.0)	13.2 (11.6 - 14.8)	0.492	14.0 (12.1 - 15.0)	11.6 (10.0 - 13.2)	< 0.001
Peak pressure, cmH_2O**[,b]	30.6 (28.6 - 32.7)	25.5 (23.2 - 27.5)	< 0.001	28.4 (26.0 - 31.0)	24.2 (22.1 - 26.7)	< 0.001
Driving pressure, cmH_2O**[,b]	17.0 (15.8 - 18.5)	12.4 (11.0 - 13.8)	< 0.001	14.4 (12.5 - 16.6)	12.3 (10.6 - 14.8)	< 0.001
Mechanical power, J/min**[,b]	21.5 (17.9 - 25.5)	17.4 (14.7 - 20.9)	< 0.001	21.2 (18.6 - 24.5)	14.7 (13.0 - 16.0)	< 0.001
Dynamic compliance, mL/cmH_2O**[,b]	26.5 (21.9 - 30.4)	37.0 (31.4 - 44.1)	< 0.001	32.0 (26.9 - 38.9)	33.6 (27.6 - 41.2)	0.090
Total respiratory rate, mpm**[,b]	23.0 (20.6 - 25.2)	21.0 (19.3 - 23.5)	< 0.001	22.8 (20.6 - 24.8)	20.0 (18.0 - 21.4)	< 0.001
Set respiratory rate, mpm**[,b]	22.8 (20.6 - 25.2)	21.3 (19.4 - 23.6)	< 0.001	22.7 (20.7 - 25.0)	20.0 (18.3 - 22.0)	< 0.001
Minute ventilation, L/min**[,b]	10.0 (8.8 - 11.6)	9.4 (8.3 - 10.7)	< 0.001	10.3 (9.2 - 11.5)	8.3 (7.5 - 9.0)	< 0.001
FiO_2**	0.6 (0.5 - 0.7)	0.6 (0.5 - 0.7)	< 0.001	0.6 (0.5 - 0.7)	0.6 (0.5 - 0.7)	0.338
$etCO_2$, mmHg**	38.0 (33.3 - 43.7)	36.3 (32.9 - 41.3)	0.022	37.5 (33.5 - 42.8)	35.5 (31.5 - 41.3)	0.007
Rescue therapy at day 01 – no (%)						
Prone positioning	97 (40.4)	116 (28.3)	0.002	148 (34.1)	61 (30.0)	0.321

eTable 4 – Baseline patient characteristics and outcomes according to the median driving pressure or mechanical power in the first four days

	**ΔP > 15 cmH_2O (*n* = 245)**	**ΔP ≤ 15 cmH_2O (*n* = 420)**	***p* value**	**MP > 17 J/min (*n* = 443)**	**MP ≤ 17 J/min (*n* = 207)**	***p* value**
Duration, hours	9.0 (4.8 - 14.0)	8.0 (3.6 - 12.0)	0.114	8.0 (4.0 - 13.0)	8.0 (3.5 - 12.0)	0.629
Recruitment maneuver	5 (2.6)	6 (1.8)	0.754	8 (2.3)	3 (1.8)	0.999
ECMO	0 (0.0)	0 (0.0)	0.999	0 (0.0)	0 (0.0)	0.999
Use of NMBA	78 (32.0)	103 (24.6)	0.047	131 (29.7)	45 (21.8)	0.037
Hours of use of use	0.0 (0.0 - 8.0)	0.0 (0.0 - 0.0)	0.055	0.0 (0.0 - 8.0)	0.0 (0.0 - 0.0)	0.018
Clinical outcome						
28–day mortality	80 (32.7)	105 (25.0)	0.039	143 (32.3)	37 (17.9)	< 0.001

Data are median (quartile 25% – quartile 75%) or No (%). Percentages may not total 100 because of rounding

CT: computed tomography; PEEP positive end expiratory pressure; ECMO: extracorporeal membrane oxygenation; FiO_2: inspired fraction of oxygen; PEEP: positive end–expiratory pressure; NMBA: neuromuscular blocking agent

* Most recent measurement in 24 hours before intubation, or at ICU admission under invasive ventilation.

** Aggregate as the mean of a maximum of four values.

*** Worst value of four available.

**** Collected in the period after intubation or ICU admission with ventilation until 24:00.

[a] Assisted ventilation defined as any mode other than pressure or volume controlled. The mode of ventilation reported is the mode used 1 hour after intubation.

[b] Only assessed in moments without spontaneous breathing activity.

eFigure 1–Distribution of Values of Driving Pressure and Mechanical Power Over the First Four Days of Ventilation

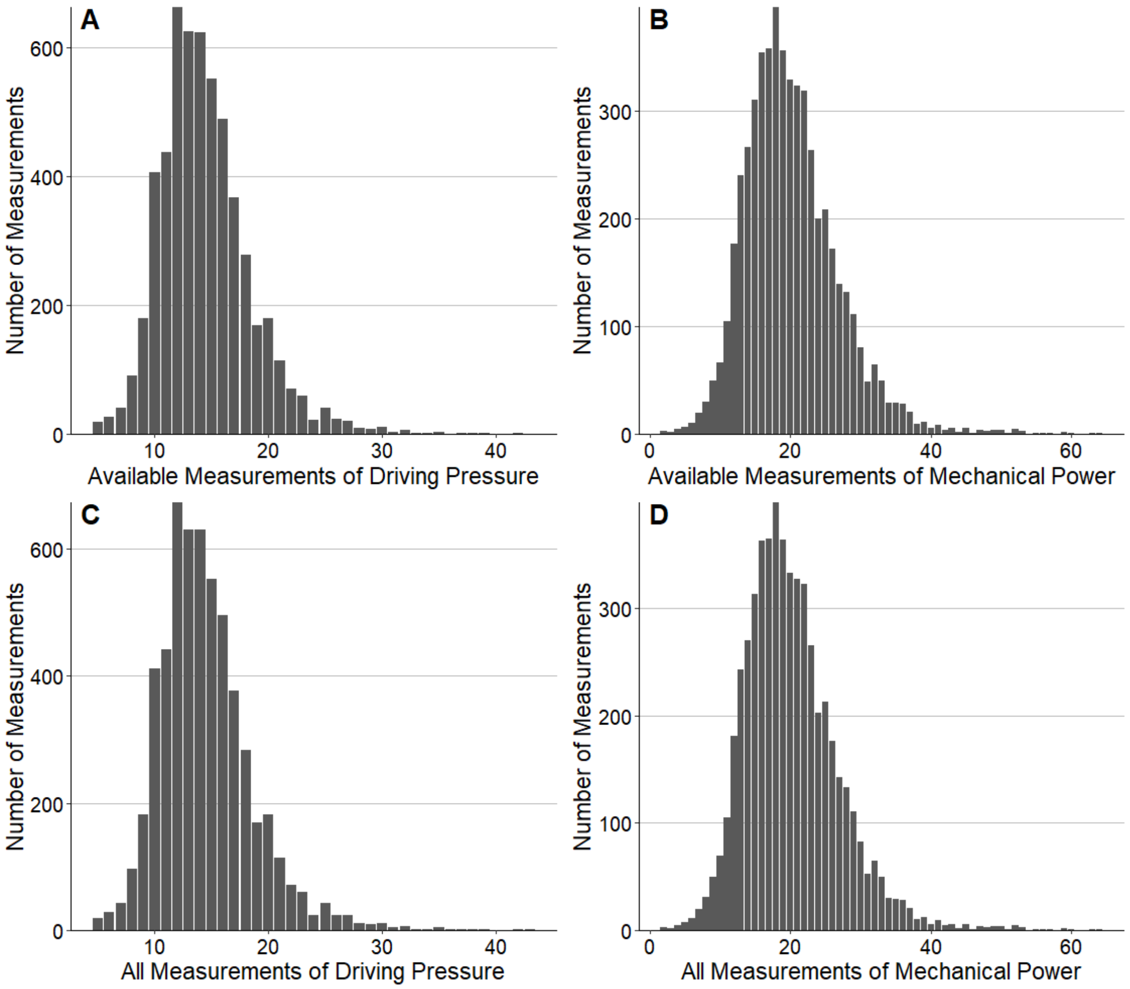

Upper panels (A and B): available measurements from the original data collection. Bottom panels (C and D): all measurements available, including those imputed by linear imputation. Both variables were calculated using only measurements without spontaneous breathing activity. When ΔP or MP were missing, a linear imputation method was used to impute values with a limit of 16 hours between each measurement available. When the gap between available measurements exceeded 16 hours, the value was considered missing.

eFigure 2–Flowchart of Included Patients

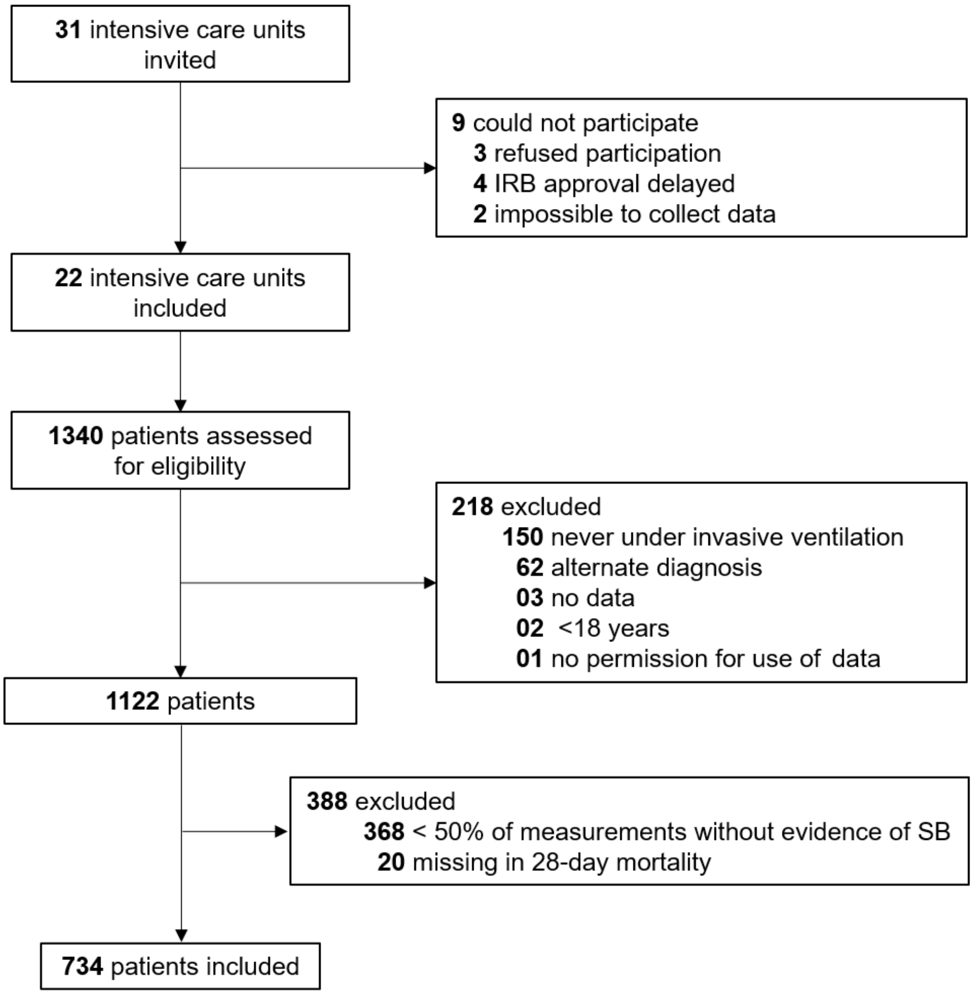

IRB: Institutional Review Board; SB: spontaneous breathing

eFigure 3–Effect of Time-Varying Driving Pressure and Mechanical Power on 28-Day Mortality According–Severity of Hypoxemia at Baseline

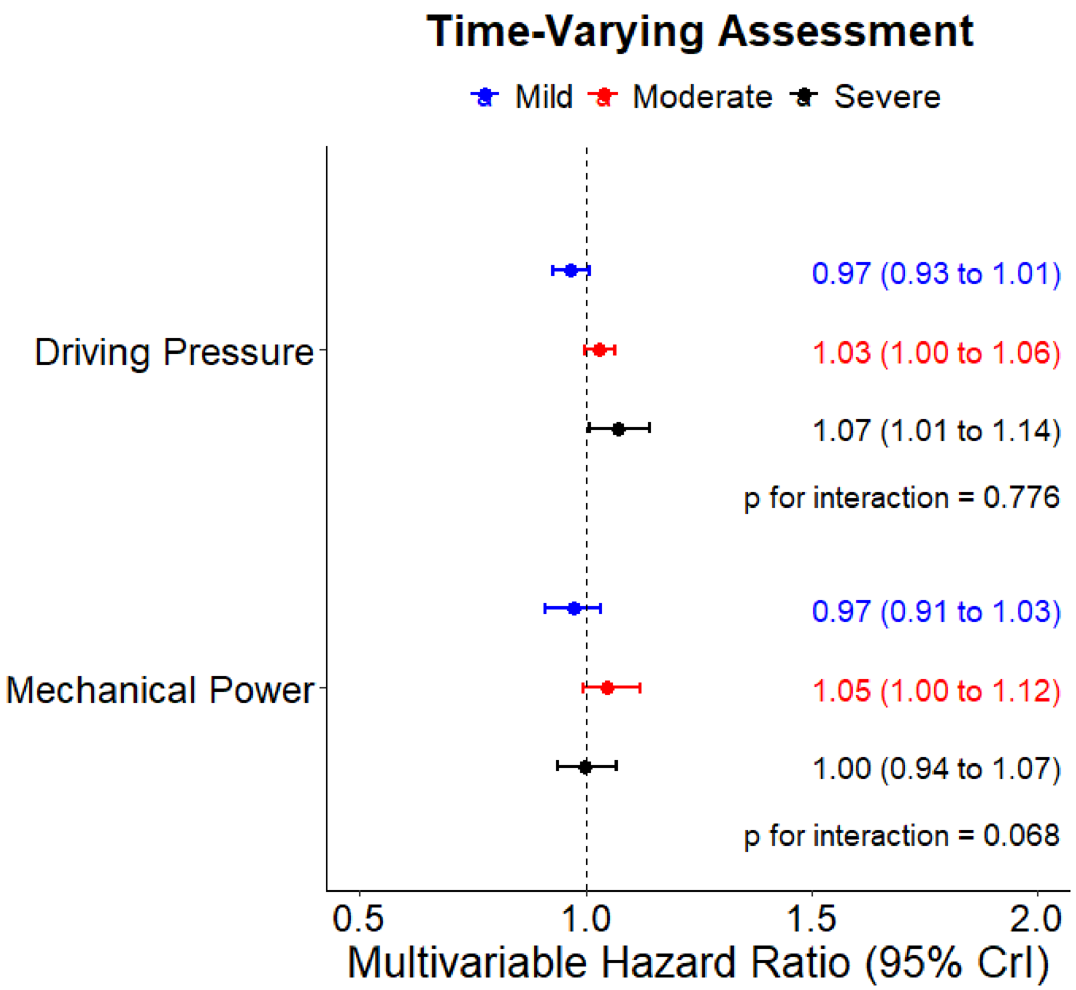

P values for interaction calculated as the tail probabilities using the formula 2 x in {P(θ > 0), P(θ < 0)}, with θ denoting the corresponding regression coefficient from the survival submodel.

eFigure 4–Number and Percentage of Measurements per Patient Above the Pre-Defined Thresholds

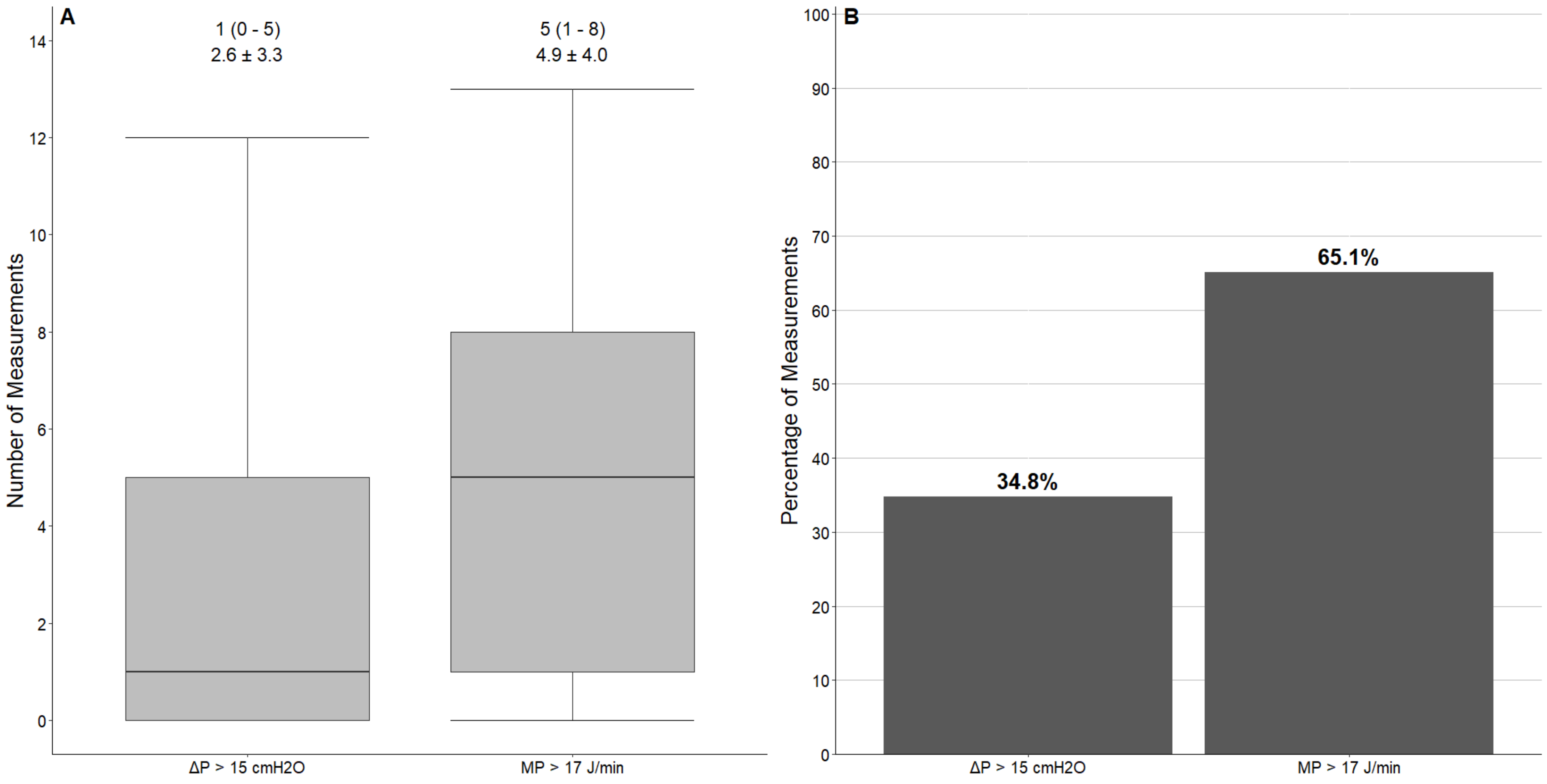

A, number of measurements per patient above the pre-defined thresholds. Numbers are median (quartile 25%–quartile 75%) and mean ± standard deviation. B, Percentage of available measurements above the pre-defined thresholds. The data reported is the mean percentage for all patients.

Boxes represent median and interquartile range. Whiskers extend 1.5 times the interquartile range beyond the first and third quartiles per the conventional Tukey method. ΔP denotes driving pressure and MP mechanical power.

eFigure 5–Time-Weighted Average and Cumulative Dose of Driving Pressure and Mechanical Power

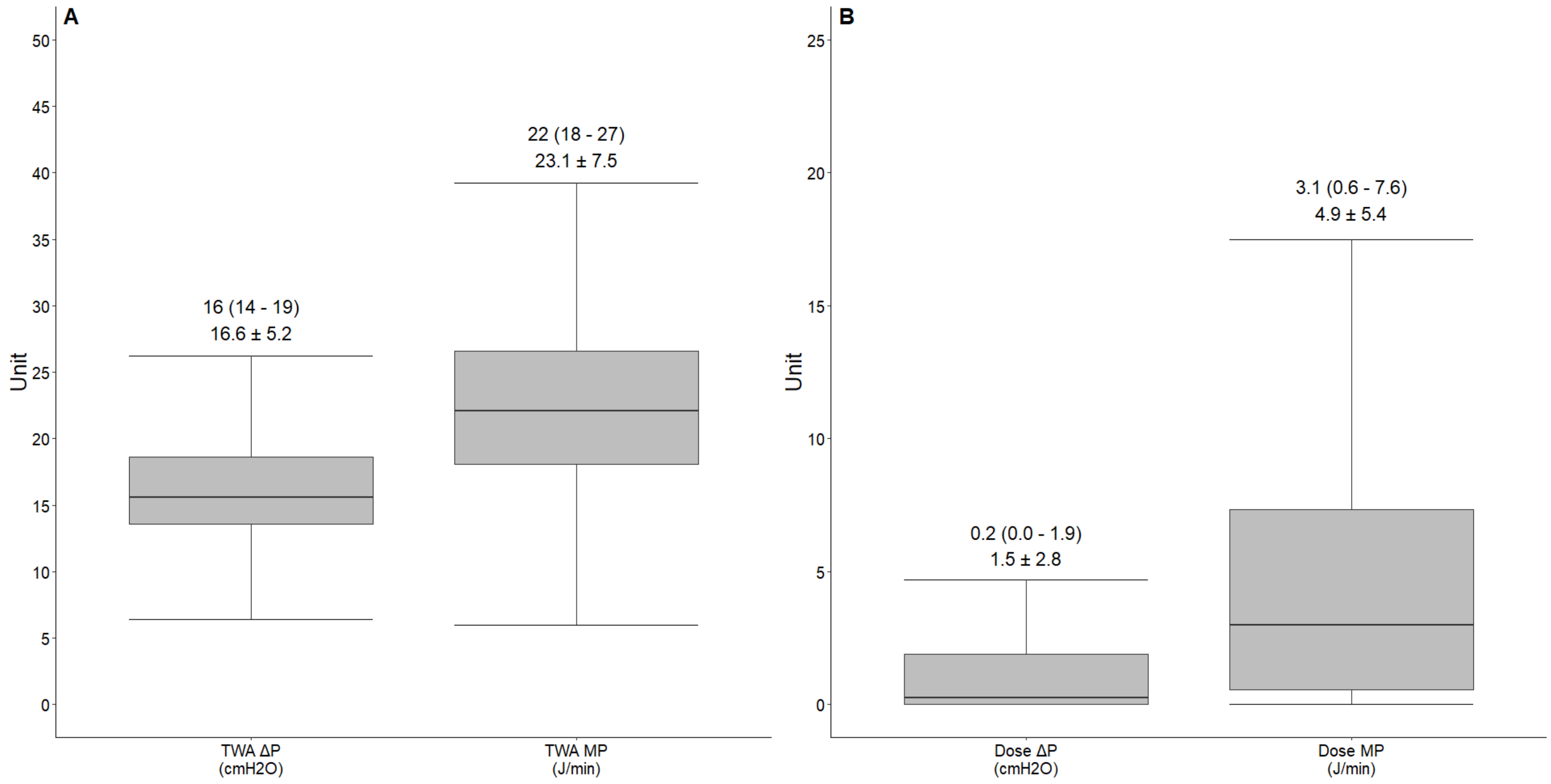

A, time-weighted average ΔP and MP. B, Cumulative dose of ΔP > 15 cmH_2O and MP > 17 J/min. Numbers are median (quartile 25%–quartile 75%) and mean ± standard deviation. Boxes represent median and interquartile range. Whiskers extend 1.5 times the interquartile range beyond the first and third quartiles per the conventional Tukey method. ΔP denotes driving pressure and MP mechanical power.
